# Post-Stroke Rehabilitation of Distal Upper Limb with New Perspective Technologies: Virtual Reality and Repetitive Transcranial Magnetic Stimulation—A Mini Review

**DOI:** 10.3390/jcm12082944

**Published:** 2023-04-18

**Authors:** Onika Banduni, Megha Saini, Neha Singh, Debasish Nath, S. Senthil Kumaran, Nand Kumar, M. V. Padma Srivastava, Amit Mehndiratta

**Affiliations:** 1 Centre for Biomedical Engineering, Indian Institute of Technology Delhi (IITD), New Delhi 110016, India; 2 Department of Nuclear Medicine and Resonance, All India Institute of Medical Sciences (AIIMS), New Delhi 110029, India; 3 Department of Psychiatry, All India Institute of Medical Sciences (AIIMS), New Delhi 110029, India; 4 Department of Neurology, All India Institute of Medical Sciences (AIIMS), New Delhi 110029, India; 5 Department of Biomedical Engineering, All India Institute of Medical Sciences (AIIMS), New Delhi 110029, India

**Keywords:** stroke, virtual reality, repetitive transcranial magnetic stimulation, distal upper limb, neuro-rehabilitation

## Abstract

Upper extremity motor impairment is the most common sequelae in patients with stroke. Moreover, its continual nature limits the optimal functioning of patients in the activities of daily living. Because of the intrinsic limitations in the conventional form of rehabilitation, the rehabilitation applications have been expanded to technology-driven solutions, such as Virtual Reality and Repetitive Transcranial Magnetic Stimulation (rTMS). The motor relearning processes are influenced by variables, such as task specificity, motivation, and feedback provision, and a VR environment in the form of interactive games could provide novel and motivating customized training solutions for better post-stroke upper limb motor improvement. rTMS being a precise non-invasive brain stimulation method with good control of stimulation parameters, has the potential to facilitate neuroplasticity and hence a good recovery. Although several studies have discussed these forms of approaches and their underlying mechanisms, only a few of them have specifically summarized the synergistic applications of these paradigms. To bridge the gaps, this mini review presents recent research and focuses precisely on the applications of VR and rTMS in distal upper limb rehabilitation. It is anticipated that this article will provide a better representation of the role of VR and rTMS in distal joint upper limb rehabilitation in patients with stroke.

## 1. Introduction

Stroke is one of the leading causes of disability, constituting 85% of upper-limb (UL) impairments and physical disability [1,2]. Post-stroke, impaired motor control leads to flaccid paresis immediately after the onset of stroke and muscle spasticity in sub-acute and chronic stages, making the performance of daily tasks difficult, leading to reduced quality of life [3]. Distal upper extremity (UE) contributes to 60% of physical upper-limb functioning. Hand function, in particular, which is imperative for performing activities of daily living (such as using a telephone or computer, turning a doorknob or key, and writing), which are strongly associated with the quality of life in stroke survivors, implying the criticality of distal joint function in post-stroke rehabilitation [4,5].

Among the rehabilitation strategies and therapeutic approaches, efforts in recent decades have increasingly focused on innovative Virtual Reality (VR) tools and non-invasive brain stimulation. VR is thought to be a promising treatment therapy, and it shows characteristics, such as high intensity, large doses of task-related training, and engaging repetitions, which may facilitate functional recovery by providing interactive tasks carried out in a computer-generated virtual environment. Additionally, it includes both visual and auditory feedback, which has been identified as crucial for motor rehabilitation [1,6]. Meta-analysis results showed that VR therapy could be effective for regaining UE motor function and activity of daily living after stroke when used in combination with conventional therapy, particularly improvement in Fugl–Meyer Assessment for Upper Limb (FMA-UL) and quality of life [2]. Any activity designed to be therapeutic at the impairment, activity, or participation levels that did not have the use of VR has been considered conventional therapy [5,7,8,9] (more details in Supplement Table S1).

Motor dysfunction restoration is a complex process involving the ability of the individual to act for the successful completion of the activity to pre-insult task levels and functional reorganization of the neural tissue [10]. The downregulation of surviving neurons and the loss of neuronal function caused by the vascular insult after stroke leads to hemiparesis [11]. Due to a decrease in neuroplasticity, post-stroke recovery is noticeably diminished over the second month and stabilizes around the sixth month [12]. Conventional therapy focuses on the active movement of the impaired arm without paying significant attention to the variations in the brain-network dynamics [13]. However, it has already been observed that repetition, intensity, and dose in traditional rehabilitation settings are insufficient to produce a plasticity-based optimal motor recovery [14]. Furthermore, patients’ participation may be limited by complex and diverse post-stroke impairments, their ability to engage in physical activity, their socio-economic background, and the degree of disability caused by the cerebrovascular event. Efforts to standardize and optimize care may be hampered by these variances within the patient population [6].

The introduction of new and effective treatment approaches was encouraged by the inherently limited scope of conventional rehabilitation settings. Identifying the pathological cortical network abnormalities and creating individualized treatment strategies to address network-level changes in neural activity may boost the chances for functional recovery [11]. Moreover, in light of the theoretical framework, multi-target, multimodal progressive repetitive interventions are required for improved sensorimotor-circuit reconstruction [6].

Repetitive Transcranial Magnetic Stimulation (rTMS) is a non-invasive brain stimulation technique that modulates cortical excitability, optimizes brain plasticity and enhances the effect of training in stroke survivors, and can act as a complementary treatment facilitating better recovery. Cortical excitability is controlled by the balance of excitatory (glutamate) and inhibitory (GABA) neurotransmission in a healthy individual. It has been hypothesized that excessive inhibition following stroke may be related to a combination of dysregulation of intrinsic GABA interneurons and disruption in interhemispheric inhibition transmitted through crossed callosal fibers where an overactive contralesional hemisphere suppresses the activity of lesioned hemisphere. The application of rTMS is aimed towards increasing cortical excitability within the ipsilesional hemisphere using high-frequency rTMS and inhibiting the contralesional hemisphere using low frequency. Studies [12,13,14] have shown an increase in cortico-motor excitability using high-frequency excitatory (>1 Hz) rTMS applied over the ipsilesional hemisphere. Several randomized controlled trials [15,16] have utilized low-frequency rTMS that can decrease the excitability of the unaffected hemisphere to improve the motor deficits within lesioned hemisphere by reducing interhemispheric inhibition (IHI) from the contralateral side [17] The ability of rTMS to alter long-term potentiation (LTP) and long-term depression (LTD) mechanisms makes it advantageous for motor learning and the neuroplasticity [17,18]. Studies have shown that low-frequency inhibitory (<1 Hz) rTMS applied across the contralesional hemisphere improves hand function [19,20] reach-to-grasp movements [21] and short-term improvement in hand dexterity, linked with a reduction in ‘Transcallosal Inhibition’ to the ipsilesional-M1 [22], with the improved ipsilesional supplementary motor area (SMA) and M1 connectivity [21,23].

rTMS has emerged as a potential exogenous neuromodulation technique attenuating cortical reorganization. Similarly, there are endogenous neuromodulation techniques, such as VR, that rely on the subject’s ability to control their brain activity and are thought to have more extensive subcortical effects. Post-stroke UL motor improvement remains sub-optimal and inconclusive, making it essential to evaluate the efficacy of VR and rTMS in combination to enhance the post-stroke UL motor improvement [24]. However, the majority of studies using VR and rTMS have been used as a rehabilitation method focused on proximal-UE, with limited information on the distal-UE [2]. As evidence suggests, both VR and rTMS contribute to improving the functional integrity of the ipsilateral–corticospinal tract (CST) and UL motor recovery in stroke survivors. However, to the best of our knowledge, there is no review of the literature to date providing insightful knowledge on successful clinical applications of combined VR and rTMS for distal-UL motor rehabilitation post-stroke and the neural mechanism underlying it. Hence, this review paper aimed to determine whether the combined effect of these technology-driven therapies outweighs their independent effects and if the combination is promising and contributes to the consolidation of rehabilitative-treatment effects.

## 2. Methods

### 2.1. Search Strategy

A search was concluded in the following search engines: MEDLINE and Google Scholar, for wider coverage of published material. The key search terms for VR distal-specific studies were (Virtual reality) AND (distal upper limb) AND (stroke) AND (rehabilitation). Key search terms for rTMS distal-specific studies were (repetitive transcranial magnetic stimulation) AND (distal upper limb) AND (stroke) AND (rehabilitation). Key search terms for combined VR and rTMS distal-specific studies were (combined) AND (virtual reality) AND (rTMS) AND (stroke) AND (rehabilitation) AND (wrist) AND (fingers). The search process included articles from the last twenty years and was finished in December 2022.

### 2.2. Study Selection

Studies were included if they were written in English, treated patients who were diagnosed with a stroke, and used either VR or rTMS (both high and low frequency) or combined VR and rTMS as the primary intervention for rehabilitation. Studies were excluded if they did not use VR or rTMS as the main intervention, such as using robotics integrated with VR therapy, haptics integrated with VR, transcranial direct current stimulation combined with VR, studies that did not focus on distal upper limb rehabilitation, studies which used transcranial direct current stimulation as main intervention and did not have at least one motor outcome.

### 2.3. Study Result

The search yielded 18,310 (MEDLINE 10 articles and Google Scholar 18,300 articles) results for VR distal specific studies, results for rTMS distal specific studies 15,504 (MEDLINE 1304 and Google Scholar 14,200), and results for combined VR and rTMS distal specific studies is 735 (MEDLINE 0 and Google Scholar 735).

Table 1 for VR-based studies in the distal upper limb, after removing 6 duplicates, 18,304 articles were reviewed. Of these articles, about 330 were selected for review of titles and abstracts based on relevance, language, and intervention (distal specific). Out of these, 51 were selected for a full-text review. The list was then narrowed down to a final list of 21 papers, as presented in Table 1.

Table 2 for rTMS-based studies in the distal upper limb, after removing 439 duplicates, 15,065 articles were reviewed. Of these articles, about 262 were selected for review of titles and abstracts based on relevance, language, and intervention (distal specific). Out of these, 20 were selected for full-text review. The list was then narrowed down to a final list of 9 papers, as presented in Table 2.

Table 3 for combined VR and rTMS-based studies in the distal upper limb, 735 articles were reviewed. Of these articles, about 23 were selected for review of titles and abstracts based on relevance, language, and intervention (distal specific). Out of these, 7 were selected for full-text review. The list was then narrowed down to a final list of 4 papers, as presented in Table 3.

## 3. Virtual Reality Therapy System

The basis of VR protocols in stroke rehabilitation is the latent ability of the brain for neuroplastic reorganization that allows motor skill acquisition, despite neurovascular insult to the brain [25] VR efficacy is increased by adding a new dimension to the neuro-feedback immersion [12]. The utilization of VR and conventional therapy are extensively compared in studies on stroke rehabilitation. A study by Laver et al. [26] found non-significant results of VR therapy over conventional therapy for improving UL function. However, they concluded that VR might be useful for UL improvement when used as a supplement to usual care. Another study by Bui et al. [6] showed no significant difference in outcomes during combined VR and conventional therapy training than conventional therapy alone for the dose-matched therapy. However, few other studies compared VR training with conventional therapy, alone as a control group for dose-matched therapy, exhibiting significant improvements in the motor function [27,28]. Thus, the literature is not conclusive in the evidence for the clinical benefit.

A study by Xi et al. [1] examined the efficacy of utilizing glass-free VR training to enhance upper-limb motor function in stroke survivors, in which 12 participants received three weeks of intervention. Fugl–Meyer upper-extremity scale (FMA-UE), rTMS measurement, and motion evaluation were assessed at the baseline and after the intervention. Post-therapy VR group has shown greater improvement in FMA-UE scores than the control group. Additionally, there was a significant improvement in Motor Evoked Potential (MEP) Latency and Central Motor Conduction Time (CMCT) in the VR group than the control group, and there is a correlation between game scores and FMA-UE scores [1]. The efficacy of VR training with YouGrabber (YouGrabber^®^ system, YouRehab AG, Zurich, Switzerland) to conventional therapy as a stand-alone therapy was compared in a randomized control trial, where patients were assigned to the experimental group or control group, and primary outcomes, such as Box and Block Test (BBT) [29], bimanual upper-limb function, Chedoke–McMaster Arm and Hand Activity Inventory [30], and Stroke Impact Scale (SIS) [2], were assessed. The training in the experimental group focused on the high repetitions of 5000 grasp movements (arm and finger) performed during games, which could possibly explain the subjective enhancement of strength observed in the stroke impact scale. Box and block test scores evidenced improvement in the experimental group and control groups; however, less-impaired patients showed bigger improvements in favor of the experimental group. These results might support the hypothesis that VR training could be beneficial for patients with mild impairment [31] (Table 1).

The inconsistencies can also be explained because of the discrepancies in different experimental techniques, methodologies, and duration of sessions as well. We noticed that there was no difference between the VR and conventional therapy groups in studies where patients received two to five rehabilitation sessions per week in contrast with more intensive therapy sessions with five days/week, demonstrating greater improvement for VR groups [4].

### 3.1. Types of VR Therapy

VR technology is categorized according to the level of immersion: (i) Immersive systems, where users are completely in synchronization with the simulated environment, thus improving the feel of presence and enabling users to interact through multisensory inputs, large screens, VR caves, and head-mounted displays, etc.; (ii) Non-immersive systems, where computer screens are used to display the virtual-environment-like videogames consoles and can be used as stand-alone or in combination with robotic exoskeletons, bionic gloves, and treadmills, thereby imparting additional feedback to the end-user [5].

VR-based rehabilitation therapy systems can be further classified according to their designs as follows: Commercial Video-Gaming Consoles (CVGC) and Custom-built Virtual Environment (CBVE) [13], such as Nintendo Wii balance board (Nintendo Co Ltd., Minami-ku Kyoto, Japan) or Xbox Kinect (Microsoft, Washington, DC, USA), a kind of non-immersive CVGC designed for recreation purposes [13]. Commercial video gaming console systems are widely used as an adjunct to standard rehabilitation for UL training as they are easy to use, enjoyable, inexpensive, and readily available. Despite the advantage of commercial video gaming console systems as a therapeutic tool for stroke, a few drawbacks associated with it have been identified, i.e., the levels of task difficulty are not readily customizable as per the requirements and capabilities of patients as they are typically designed for healthy individuals, which, in turn, is excessively challenging for stroke survivors. Additionally, multiple environmental factors are not taken into account, which might have beneficial effects on UL functional recovery [13].

On the other hand, clinicians and researchers designed custom-built virtual environment systems to enhance the patient’s sense of presence, provide patient-centered training, aid the automatic tracking of patient’s movements, and reduce the excessive use of unwanted compensatory movements [14]. Although preliminary evidence exists in support of the better effectiveness of custom-built virtual environments [28], its actual effect is still inconclusive [5].

Gamified rehabilitation refers to the use of game-like elements (e.g., points, rewards, and challenges) and has been extensively used in the literature with the term “Non-immersive VR”. Immersive VR is a new emerging experience provided using various VR headsets, such as Oculus, that can track users’ movements to create a more realistic experience. The main difference between gamified rehabilitation and VR is the level of immersion and interactivity that each technology provides. However, considering the associated high cost, the practical implementation of immersive VR might be challenging for individual patients or resource-limited clinical settings and, thus, is still under research. Cognitive decline is a well-established association with stroke; immersive VR-based solutions require a pre-requite cognitive function for understanding and usage of this complex solution. Non-immersive VR solutions have been a viable option in terms of cost, the ability to be integrated with various easily accessible interfaces [26,32,33], having a minimal chance of virtual motion sickness when used for a prolonged period [33,34] and are easy to execute in stroke survivors with cognitive dysfunction.

**Figure 1:**
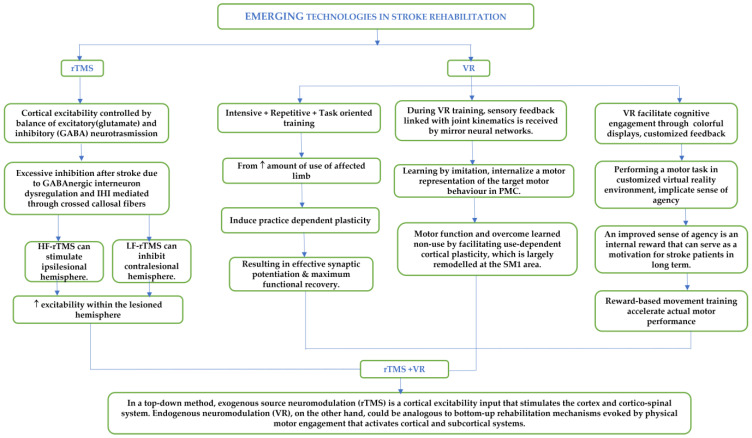
Combined neurophysiological effects of VR and rTMS. HF: High Frequency; LF: Low Frequency; PMC: Primary Motor Cortex; rTMS: Repetitive Magnetic Stimulation; SMC: Primary Sensorimotor Cortex; VR: Virtual Reality.

The motor relearning processes are influenced by variables, such as variability, task specificity, motivation, and feedback provision [15], and could be integrated with the VR environment [26,32] in the form of interactive games, which can help provide novel and motivating customized training solutions for better post-stroke UL motor improvement by limiting fatigue, loss of enthusiasm, and improving cooperation [31]. Task practice in VR environments with feedback can enhance neuroplasticity in stroke survivors, leading to activation in the ipsilesional hemisphere and facilitating motor relearning of UL tasks. The criticality of feedback has been well established in the field of neuro-rehabilitation, contributing to domains of skill acquisition and mediating use-dependent plasticity (UDP) along with completing sensorimotor loops [35]. Although neural mechanisms underlying practice-dependent motor recovery are unclear, it has been hypothesized that intensive use of the paretic limb could promote effective synaptic potentiation, thereby increasing use-dependent plasticity. (Figure 1) [36].

In addition, individualization of task design, minimal supervision by therapists, and flexibility in tele and home-based use make VR a potentially advantageous form of rehabilitation [37]. VR systems with tracking functionality allow therapists to keep track of patients’ progress without requiring any continuous physical supervision. A few VR systems have been particularly designed for home usage, such as Neurofenix Ltd. (NeuroBall™ platform, London, UK) platform, with the purpose of motivating patients to exercise independently at home, in their environment and comfort, and with the least therapist supervision [38]. Few studies reported that patients had gained significant improvements in bilateral UL function, grasp strength, and motor control after four weeks of training at home [39,40]. A recent study suggested that home-based VR training can stimulate cortical reorganization and is linked with UL functional improvement [41]. All this recent evidence advocate VR as an imperative tool for the development of telerehabilitation, and the promising results strongly motivate further investigation of the home-based VR training [6].

### 3.2. Mechanisms of VR Training

Two important neurological mechanisms of VR training, involving cortical reorganization and corticospinal tract recovery, have been revealed as key contributors to the improvement of the UL motor function [42] (Figure 1). Considering Wolf Motor Function Test as a motor function measure and functional MRI to measure the neuronal activation, 4 weeks of Leap-motion (LMC^®^; Leap Motion, Inc, San Francisco, CA, USA)-based VR training in sub-acute patients demonstrated an increase in functional MRI-activation of the lesioned hemisphere and reduction in action performance time in the experimental group [27]. Pre-therapy, there was activation of bilateral or ipsilateral primary sensorimotor cortex (SMC), SMA, and cerebellum in both groups. Post therapy, intensive use of the affected limb in both groups led to a shift in SMC-activation from ipsilateral or bilateral to contralateral regions, which was more significant in the experimental group than in the control group [27]. Repetitive use of the affected limb may enhance use-induced neuroplasticity by producing an effective synaptic potentiation [27]. The greater intensity of practice may induce a shift in the SMC throughout cerebral hemispheres in the experimental group than that in the control group by use-induced neuroplasticity.

In another study, during VR training, the patient observed and mirrored the movements of the representation (avatar) by controlling the avatar through hand–arm coordination in the real world and performing the required tasks in the games produced through a virtual environment [43]. Studies have suggested that a reorganization mechanism of the imitation-dependent cortex neuroplasticity via mirror neural networks may be produced after VR training [27]. Another study used functional MRI to evaluate the neural mechanisms of UL motor recovery after four weeks of VR intervention and noticed that pre-therapy, greater bilateral motor networks were activated, although post-therapy only ipsilesional-SMC was activated, showing a shift in the cortical structure of affected limb from the ipsilateral to contralateral hemisphere [43]. Similarly, it might be possible that frequent use of the affected limb along with sensory feedback during VR training learned to internalize motor representation of target motor behavior which might aid use-dependent plasticity that can help in overcoming learned non-use and facilitate motor-function recovery. Sensory feedback and virtual reality-based augmented feedback are both used in VR-based rehabilitation settings, but they differ in several ways. As per available literature, augmented feedback is usually extrinsic in nature, while sensory feedback is usually intrinsic [44]. Intrinsic feedback is associated with the internal sensory process of an individual as an outcome of the execution of a movement, including vision, proprioception, audition, and tactile/haptic information [14,44] Augmented feedback is usually given in supplement to the intrinsic feedback to provide extra information to the internal sensors of the body (ear, skin, eye) originating from an external source (auditory, visual, or sensory) [44,45].

Stroke survivors having hemiplegia have the tendency to neglect the usage of the affected limb and develop a very poor sense of agency [46]. The sense of agency refers to the feeling of control over actions and has been found to be associated with internal motivation or reward for those actions in the long-term [47]. The provision of optimal feedback, personalization, and incentivization of progress with a reward during VR-based training not only establishes a greater commitment to the therapeutic protocol but also facilitates a greater sense of agency to accelerate actual movement performance [48]. Incorporation of such reward-based movement training has been found to expedite protocol adaptation, skill-learning, and activity-dependent plasticity [48,49] (Figure 1).

### 3.3. VR-Based Rehabilitation for Distal Upper Extremity

Recent studies on UL rehabilitation for stroke survivors found that the use of VR-based rehabilitation is more efficacious than dose-matched conventional therapy for regaining UL function [5,49]. However, the majority of studies available on VR-based UE rehabilitation focus on the proximal UE, with limited data on distal UE. Despite the fact that a few studies have demonstrated positive outcomes for VR rehabilitation of the distal UE, these trials did not involve a control group [41,42] (Table 1). The effects of VR-based rehabilitation with standard occupational therapy using a smart glove were examined on 46 stroke survivors in a randomized controlled trial for distal UE function and Health-Related Quality of Life and compared the findings to those of dose-matched conventional therapy [5] (Table 1), where experimental group demonstrated significant improvements in FMA [50] Jebsen–Taylor test (JTT-total and JTT-gross) [51], and stroke impact scale (composite and overall SIS, SIS social participation, and SIS mobility) scores than the control group [5] (Table 1). In a recent pilot study, custom VR tasks were designed for distal UE rehabilitation and validated on forty healthy subjects and two-stroke survivors for comparison. Each participant received a 90 min VR session, and task-specific performance measures, such as time taken to complete the task, relative percentage error, smoothness of trajectory, and trajectory plots, were assessed. Performance metrics assessed from healthy subjects were used as a reference for patients. The results demonstrated that the task-performance parameters helped in assessing the patient’s progression quantitatively [52].

**Table 1:** VR-based studies primarily focusing on distal upper limb.

Sl No. and Studies	Subjects	Device Used	Intervention	Outcome Measures	Joints Involved	Conclusion
1. Nath et al. [53]	1 chronic stroke survivor	Extreme 3D Pro Joystick (Logitech, Lausanne, Switzerland) (Non-immersive)	45 min/session, 5 sessions per week for 4 weeks	Clinical Scales (FMA, MAS, MBI, SIS, BS, MRS), Neurophysiological measures (fMRI, DTI, MEP), task-specific metrics	Wrist and fingers	The pilot study exhibited preliminary clinical potential of the customized VR tasks specific for distal upper limb in chronic phase of recovery
2. Fong et al. [54]	20 chronic stroke survivors	Leap Motion (LMC^®^; Leap Motion, Inc, San Francisco, CA, USA) (Non-immersive)	30 min/session, 5 sessions per week for 2 weeks	FMA-UE, WMFT, MAL	Wrist and fingers	Task-specific VR training was helpful in upper-extremity recovery in patients with chronic stroke
3. Miclaus et al. [55]	52 Stroke survivors Experimental group [6 = subacute group, 20 = chronic] Control Group [5 = subacute group, 21 = chronic control group]	MIRA software (MIRA Rehab Ltd., London, UK) for Non-immersive virtual reality (NIVR) therapy	2 weeks	FMA-UE, MRS, FIM, AROM, MMT, MAS, FRT	Wrist and Fingers	The results suggest that NIVR rehabilitation is efficient to be administered to post-stroke patients, and the study design can be used for a further trial, in the perspective that NIVR therapy can be more efficient than standard physiotherapy within the first six months post-stroke
4. Qiu et al. [56]	15 chronic Stroke survivors	Home-Based Virtual Rehabilitation (HoVRS)	15 min every weekday for 3 months	Hand Opening Range (HOR), Hand Opening Accuracy (HOA), Wrist Pitch Range (WPR), Wrist Pitch Accuracy (WPA), Hand Roll Range (HRR), Hand Roll Accuracy (HRA), FMA-UE	Shoulder, Elbow, Wrist, Hand, Whole arm	Persons with chronic stroke were able to use the system safely and productively with minimal supervision resulting in measurable improvements in upper extremity function
5. Ögün et al. [57]	33 chronic Stroke survivors	Leap Motion (LMC^®^; Leap Motion, Inc, San Francisco, CA, USA) (Non-immersive)	60 min, 3 days/week, 6 weeks (18 sessions)	ARAT, FIM, FMA-UE	All finger gestures	Immersive VR rehabilitation appeared to be effective in improving upper extremity function and self-care skills, but it did not improve functional independence
6. Ahmadi et al. [58]	30 chronic stroke survivors	VR E-Link (Biometrics Ltd., Gwent, UK)	1 h (3× per week)	FMA-UE, SIS, CAHAI, MI, MAS, MMSE and goniometer	Forearm and wrist	VR-based computer games in combination with routine occupational therapy interventions could improve upper extremities functional impairments in chronic stroke patients.
7. Kim et al. [59]	23 sub-acute stroke survivors	Kinect (Microsoft Corp., Redmond, WA, USA) (Non-immersive)	30 min/day for 10 days	BBT, FMA, BS, K-MBI, total activity count	Wrist angle, grasp	Kinect-based upper limb rehabilitation system was not more efficacious compared with sham VR. However, the compliance in VR was good, and VR system induced more arm motion than control and similar activity compared with the conventional therapy, which suggests its utility as an adjuvant additional therapy during inpatient stroke rehabilitation
8. Wang et al. [27]	26 subacute stroke survivors	Leap Motion based VR system (LMC^®^; Leap Motion, Inc, San Francisco, CA, USA) (Non-immersive)	EG were given VR training for (5× a week for 4 weeks), + OT for 45 min, (5× a week for 4 weeks). CG received conventional OT twice a day, each for 45 min, (5× a week for 4 weeks)	Primary Outcome-WMFT Secondary Outcome-fMRI	Hands and fingers	↓ Action performance time in WFMT in EG. ↑↑ Activation intensity and laterality index of contralateral primary sensorimotor cortex (both in EG and CG)
9. Standen et al. [60]	18 stroke survivors	Virtual glove (with 4 IR LEDs on finger tips), fingers tracked using Nintendo Wiimote (Non-immersive)	20 min (thrice in a day) for 8 weeks	WMFT, 9-HPT, MAL, NE-ADL	Movements of reach to grasp, grasp and release, pronation and supination	Significantly greater change from baseline in the intervention group on midpoint Wolf Grip strength and two subscales of the final MAL
10. Brunner et al. [61]	120 sub-acute stroke survivors	YouGrabber system (Non-immersive)	60 min sessions, 4 weeks	ARAT, BBT, FIM	Fingers and arm	Additional upper extremity VR training was not superior but equally as effective as additional CT in the subacute phase after stroke. VR may constitute a motivating training alternative as a supplement to standard rehabilitation
11. Shin et al. [5]	46 stroke survivors	RAPAEL Smart Glove (Neofect, Yong-in, Korea) (Non-immersive)	4 weeks (SG or CON groups) (20 sessions × 30 min/day) along with standard OT daily for 30 min	Primary outcome—FM scores, and the Secondary outcomes—JTHFT, PPT, and SIS version 3.0	Forearm, Wrist and fingers	VR-based rehabilitation combined with standard occupational therapy might be more effective than amount-matched conventional rehabilitation for improving distal upper extremity function and HRQoL
12. Tsoupikova et al. [50]	6 chronic stroke survivors	VR system with PneuGlove [62] (Immersive)	18 (1 h training sessions) with the VR system over a 6-week period	FMA-UE, FMWH, CMSA_A and CMSA_H, ARAT, BBT, Grip and palmar and lateral pinch strengths	Arm, wrist, hand	↑ lateral pinch strength
13. Brown et al. [63]	9 chronic stroke survivors	Neuro game therapy video game [64] (Non-immersive)	45 min, 5 times a week, 4 weeks	WMFT, CAHAI, pre-post EMG measures	Wrist ROM	use of the electromyography-controlled video game impacts muscle activation. Limited changes in kinematic and activity level outcomes
14. Schuster-Amft et al. [65]	60 chronic stroke survivors	YouGrabber system, G*Power (Non-immersive)	16 sessions (45 min each), 4 weeks	BBT, CMSA, SIS, MBI, MMSE	Finger and wrist	Study Ongoing
15. Merians et al. [66]	12 stroke survivors	CyberGlove (Immersion Corporation, San Jose, CA, USA) and CyberGrasp (Immersion Corporation, San Jose, CA, USA) (Immersive)	4 UE gaming simulations (4×/day,2weeks) Training on day 1 (2–3 h) along with 15 min increments during 1st Week, up to 3 h in Week 2	Primary outcome—WMFT and JTHFT Secondary outcome—kinematic measures obtained from the Hammer task and the Virtual Piano	Hand and fingers	Complex gaming simulations interfaced with adaptive robots requiring integrated control of shoulder, elbow, forearm, wrist, and finger movements appear to have a substantial effect on improving hemiparetic hand function
16. Proffitt et al. [67]	1 chronic stroke survivor	Nintendo wii remotes (Non-immersive)	5 days/week for 6 weeks, 60–75 min each day	ARAT, ACS, RPS	Shoulder, elbow and wrist flexion and extension	Results indicate that computer games have the potential to be a useful intervention for people with stroke
17. Yavuzer et al. [68]	10 sub-acute stroke survivors	eyetoy playstation (Sony, Tokyo, Japan) game (Non-immersive)	5 days/week, 4 weeks, 2–5 h/day	BS, FIM	Flexion and extension of paretic shoulder, elbow, wrist, abduction of shoulders	“PlayStation EyeToy” Games combined with a CT have a potential to enhance upper-extremity-related motor functioning in sub-acute stroke patients
18. Merians et al. [69]	8 chronic stroke survivors	Cyberglove (Position), RMII Force feedback glove [70] (Non-immersive)	3 weeks, 2–2.5 h/day	JTHFT	Fingers	Transfer of the improvements was demonstrated through changes in the JTHFT and a decrease after the therapy in the overall time from hand peak velocity to the moment when an object was lifted from the table
19. Adamovich et al. [71]	8 stroke survivors	Cyberglove, RMII glove, EM position trackers (Non-immersive)	2–2.5 h/day, 13 days	JTHFT	Finger	Improved JTT on transfer of motor learning to real world tasks
20.Boian et al. [72]	4 stroke survivors	Cyberglove, RMII glove (Non-immersive)	2 h/day, 5 days/week for 3 weeks	JTHFT	Thumb and finger	Gain in thumb range, finger speed, fractionation, good retention, improved JTT, faster grasping
21. Jack et al. [73]	3 stroke survivors	Cyberglove and (RMII) force feedback glove (Non-immersive)	Conventional rehab+ VR, 9 daily sessions (5hr each)	Hand movement, Range, Speed, Fractionation, Strength	Hand	Thumb ROM, angular speed (improved), fractionation improved, approx. session’s mechanical work capacity improved, improved grasping force, +changes in Jebsen hand score

Abbreviations: 9-HPT: Nine Hole Peg Test; ACS: Activity Card Sort; ARAT: Action Reach Arm Test; BBT: Box and Block Test; BS: Brunnstrom stage; CAHAI: Chedoke Arm and Hand Inventory; CG/CON: Control Group; CMSA: Chedoke–McMaster Stroke Assessment; CMSA_A: Chedoke–McMaster Stroke Assessment Stage of Arm; CMSA_H: Chedoke–McMaster Stroke Assessment Stage of Hand; EG: Experimental Group; FIM: Functional Independence Measure; FMA-UE: Fugl–Meyer Assessment-Upper Extremity; fMRI: Functional MRI; FMWH: Fugl–Meyer Assessment for the Wrist and Hand; FRT: Functional Reach Test; HRQoL: Health-Related Quality of Life; JTHF/JTT: Jebsen Test of Hand Function; JTHFT: Jebsen–Taylor Hand Function Test; K-MBI: Korean Version of Modified Barthel Index; MAL: Motor Activity Log; MBI: Modified Barthel Index; MMSE: Mini-Mental State Examination; MMT: Manual Muscle Testing; MRS: Modified Ranking Scale; NE-ADL: Nottingham Extended Activity of Daily Living; PPT: Purdue Pegboard Test; RPS: Reaching Performance Scale; rTMS: Repetitive Transcranial Magnetic Stimulation; SIS: Stroke Impact Scale; VR/VE: Virtual Reality/Virtual Environment, WMFT: Wolf Motor Function Test.

The effects of the VR system using Leap Motion were studied with 26 subacute stroke survivors in the experimental group and control group. The four weeks of treatment exhibited considerable improvements in motor functions with better improvements in the experimental group, as evidenced by a reduction in Wolf Motor Function Test action performance time [27]. The effect of Leap Motion-based VR training is related to the idea of high-intensity, repetitive, and task-orientated training. Hence, the high-intensity practice seems to promote better outcomes in the event of impairments [27] (Table 1). Most of the studies focused on distal UL have a small sample size, but the results suggest that VR-based rehabilitation might be effective when combined with conventional therapy for improving distal UL function [5] (Table 1). Therefore, further studies are required on a larger cohort of patients with follow-up for evaluating any long-term benefits.

An immersive HTC-VIVE head-mounted display (HTC Corp., Taiwan) was used to provide unilateral and bilateral UE training to 23 stroke survivors, in which the VR group revealed significant improvements in FMA-UE scores to the control group [74]. Neural activity increased post-intervention, particularly in the brain areas implicating mirror neurons, such as in primary SMC. Another head-mounted display-based study showed that fully-immersive VR training might improve UE function, as evidenced by improvement in Action Research Arm Test (ARAT) and Box and Block Test, with no reported adverse events [75]. Although a head-mounted display improves the depth of perception compared to a 2D flat-screen display, other feasible strategies needed to enhance VR efficacy include object occlusion, lighting/shadow effects, colour shading, and relative scaling of objects by taking depth, perspective projection, and motion parallax [76]. User experience obtained while performing tasks using a head-mounted display and without a head-mounted display was examined and concluded with no relevant differences while performing using a head-mounted display [77]. Despite the promising literature, there is still inconclusive evidence of the transference of skills achieved via VR intervention to real-life settings, and it needs further investigation [76].

## 4. Transcranial Magnetic Stimulation Therapy

### 4.1. Mechanism of Modulation of Cortical Excitability with Repetitive Transcranial Magnetic Stimulation (rTMS)

It is widely evidenced that high-frequency rTMS (≥5 Hz) produces an increase in cortical excitability through a long-term potentiation mechanism, which involves stimulation of presynaptic neurons followed by stimulation of postsynaptic neurons [27,74] and also has been described to enhance reaction-time performance and motor sequential learning task performance [22]. Low-frequency rTMS (≤1 Hz, LF-rTMS) has been shown to inhibit cortical excitability through a long-term depression mechanism with stimulation of postsynaptic neurons followed by presynaptic neurons [77,78]. Using inhibitory rTMS over the unaffected hemisphere has been evidenced to be superior and showed uniform results than facilitating rTMS over the affected hemisphere, possibly because the unaffected site is unlikely to be affected by neuronal loss or tissue damage. Therefore, the neuro-modulatory effects of 1-Hz rTMS on the unaffected hemisphere might be superior to that of 3-Hz rTMS on the affected hemisphere for the stroke recovery [79]. However, it is still debatable whether an application of high-frequency rTMS to the ipsilesional hemisphere, causing cortical excitability, or low-frequency rTMS to the contralesional hemisphere, causing inhibition in the cortex, would be more effective in enhancing post-stroke motor recovery [80]. Nevertheless, as described, rTMS might be a safe and minimal-risk non-invasive brain stimulation modality with better translational potential from research to the mainstream rehabilitation setup [81]. Despite the lack of standardized operating methods and harmonization, the application of rTMS has been found to be a promising technique for stroke rehabilitation. However, a better knowledge of the underlying mechanics and protocol standardization should be encouraged [82].

The dorsal premotor cortex in the contralateral hemisphere may be facilitated by low-frequency rTMS (LF-rTMS) over contralesional-M1. Many studies have recommended that the recovery of motor function post-stroke is due to the dorsal premotor cortex activity in the ipsilesional hemisphere. Hence, it is possible that the activity of ipsilesional- dorsal premotor cortex induced by rTMS over contralesional-M1 leads to functional improvement [83]. Sixty-nine stroke survivors in a randomized controlled trial underwent five daily sessions of 3-Hz ipsilesional rTMS, 1-Hz contralesional rTMS, or sham rTMS in addition to conventional therapy. The effects of HF versus LF-rTMS on motor recovery during the early stage of stroke were compared, and neurophysiological correlates of motor improvements were determined. FMA-UL, Muscle Research Council Scale, Barthel Index, and Modified Rankin Scale, along with measures of cortical excitability, were obtained where the experimental group demonstrated greater motor improvements than the control group, which lasted for at least three months [79]. Low-frequency 1-Hz rTMS over the contralesional hemisphere facilitated upper-limb motor performance more profoundly than 3-Hz rTMS on the ipsilesional hemisphere. A significant correlation between improved motor function and changes in motor-cortex excitability in the affected hemisphere was also observed [79]. Numerous studies have exhibited improvements in hand function and reduction in spasticity with the application of rTMS (Table 2).

**Table 2:** rTMS-based studies primarily focusing on distal upper limb.

Sl No. and Studies	Participants	Muscle Involved (MEP)	Intervention	Outcome Measure	Findings
1. Askin et al. [84]	40 chronic stroke survivors	Index Finger Flexion	2 groups: rTMS Group- LFrTMS-1Hz, 1200 pulses with an intensity of 90% of RMT were delivered to the unaffected hemisphere for 20 min. Each patient received a total of 10 sessions in 2 weeks (5 days/week) before PT sessions; CON group: 20 session of PT (5 days/week × 4 weeks)	BRS, UE-FMA, BBT, MAS, FIM scale, MMSE, and FAS.	↓ Distal and Hand MAS score significantly ↑ FMA-UL, BBT, FIM, FAS, FIM cognitive score, MMSE score in both LF-rTMS and CON groups; these changes were significantly greater in the rTMS group.
2. Saadati et al. [17]	24 sub-acute stroke survivors	Thenar muscle	3 groups: HF-rTMs (10 Hz); LF-rTMS (1 Hz); Routine Rehab (3× a week for 10 sessions)	WFMT and Hand Grip	↓ active MEP within the group ↑ WFMT and grip test in the HF group
3. Wang et al. [85]	44 stroke survivors (3 to 12 months following stroke)	FDI muscle	3 groups: cPMD(dorsal premotor cortex); cM1(primary motor cortex); Sham Each received 10 session of 1-Hz rTMS	MRC, FMA, WFMT	cPMd modulation yielded significant improvements in MRC, FMA, and WMFT scores compared with sham stimulation and a significant effect on cortical excitability suppression equivalent to that of cM1 modulation, but engendered effects on motor improvement inferior to those of cM1 modulation
4. Galvão et al. [86]	10 in rTMS group, 10 in sham stroke survivors	FDI muscle	10 sessions of rTMS	MAS, FMA-UE, maximum PROM of the paretic wrist joint, FIM	MAS decreased with rTMS
5. Sung et al. [87]	54 sub-acute stroke survivors (15 group A: 1-Hz + iTBS, 12 group B: sham 1-Hz + iTBS, 13 group C: 1-Hz + sham iTBS, 14 group D: sham 1-Hz + sham iTBS)	FDI muscle	20 sessions	WMFT, FMA—UE, finger flexor MRC, index FTT	MRC, FMA, WMFT, FTT, and RT showed significantly greater improvement in patients who experienced real stimulation
6. Kakuda et al. [88]	39 chronic stroke survivors	FDI muscle	22 sessions of LF-rTMS applied to the non-lesional hemisphere and OT (one-to-one training and self-training)	MAS, WMFT, FMA-UE	Decrease in MAS for wrist and finger, increase in FMA-UE and lesser WMFT performance time
7. KoganEmaru et al. [89]	9 chronic stroke survivors	EDC muscle	1 exercise + rTMS (Eex-TMS) + 1 exercise + sham (Eex) + 1 rest + rTMS (TMS) (each session on separate days) at 5 Hz, 15 cycles (15 min), each cycle: 50 s exercises/rest + 1 s rest + 8 s rTMS (40 pulses)/sham + 1 s rest, (SM) sham coil	AROM and PROM, pinch force, grip power and MAS	Active range of movement was significantly increased in extension for the wrist joint, thumb, index, and middle finger MCP joint by “TMS” session
8. Takeuchi et al. [83]	20 chronic stroke survivors	First Dorsal Interosseous (FDI)	2 groups: Sham vs. Real rTMS (10 in each group) and received rTMS at contralesional M1(1 Hz, 25 min)	Pinch force and acceleration, RMT, MEP amplitude, and TCI duration	↓ amplitude of MEP in contralesional M1 and TCI duration (rTMS group) rTMS induced improvement in pinch acceleration of the affected hand
9. Boggio et al. [23]	1 chronic stroke survivor	Abductor pollicis brevis muscle	A sham stimulation for 2 months and active stimulation after 2 months LF- rTMS) of on unaffected hemisphere at intensity of 100% of MT in a continuous train of 20 min, 1200 pulses. After 4 mos, the patient returned for a new session of active rTMS using the same parameters of stimulation	Thumb flexion, extension, abduction, and adduction and wrist flexion and extension were assessed before and after the treatment.	Significant improvement in motor function after active, but not after sham stimulation of the unaffected primary motor cortex

Abbreviations: AROM: Active Range of Motion; BRS: Brunnstrom Recovery Stages; BBT: Box and Block Test; EDC: Extensor Digitorum Communis; FAS: Functional Ambulation Scale; FDI: First Dorsal Interosseous; FIM: Functional Independence Measure; FMA-UE: Fugl–Meyer Assessment-Upper Extremity; FTT: Finger-Tapping Test; HF-rTMS: High-Frequency Repetitive Transcranial Magnetic Stimulation; iTBS: Intermittent Theta Burst Stimulation; LF-rTMS: Low-Frequency Repetitive Transcranial Magnetic Stimulation; MAS: Modified Ashworth Scale; MCP: Metacarpophalangeal Joint; MMSE: Mini-Mental Status Examination; MRC: Muscle Research Council Scale; PROM: Passive Range of Motion; RMT: Resting Motor Threshold; rTMS: Repetitive Transcranial Magnetic Stimulation; RT: Simple Reaction Time Task; TCI: Transcallosal Inhibition; WMFT: Wolf Motor Function Test.

### 4.2. rTMS Studies on Distal Upper Extremities

A meta-analysis on the effects of rTMS on hand function recovery and neuroplasticity in subcortical stroke reported positive effects of rTMS on finger motor-ability and hand function. However, neurophysiologic measurements (changes in motor evoked potential and active motor threshold) post-stimulation were insignificant, with few adverse effects reported [90]. In a randomized, double-blinded study of real (25 min subthreshold 1 Hz rTMS at contralesional-M1) versus sham-rTMS, the real group exhibited reduced motor evoked potential amplitudes in contralesional M1; the reduced transcallosal inhibition-duration immediately induced an improvement in pinch acceleration as compared with sham group [83]. When stroke survivors moved their affected hand, transcallosal inhibition from contralesional to ipsilesional M1 was found to be abnormally high [91]. Taking these findings into consideration, 1-Hz rTMS could be considered to improve motor function by reducing the transcallosal inhibition from contralesional-M1 to ipsilesional-M1. Moreover, rTMS over M1 has been evidenced to induce disinhibition in contralateral-M1 [92]. Reduced inhibition promotes the unmasking of functionally latent neural networks pre-existing around the lesion, thus contributing to cortical reorganization. It might be feasible that the disinhibition of affected M1, induced by disruption of transcallosal inhibition, could also contribute to the recovery promoted by unmasking of latent-networks [83] (Table 2).

Saadati et al. [17] studied the effect of rTMS along with conventional therapy to compare the effects of rTMS-protocols with conventional therapy on hand-motor functions and corticomotor excitability in stroke survivors. Twenty-four hemiplegic patients were randomly assigned to three groups: high-frequency rTMS (10 Hz); rehabilitation programs with LF-rTMS (1Hz); and routine rehabilitation programs only. Ten sessions of 20 min were given, and Motor Evoked Potential, Wolf Motor Function Test, and handgrip power (by a dynamometer) were assessed pre-test, post-test, and after an 8-week follow-up. The results demonstrated that a reduction in resting motor threshold in the experimental group receiving HF-rTMS was not statistically significant; the active motor threshold was found to be significantly reduced within the group. Furthermore, the results of the Wolf Motor Function Test and grip test were statistically significant in the HF group, indicating that HF-rTMS over the ipsilesional hemisphere combined with conventional therapy can significantly improve hand functions and neurophysiology via specifically increasing contralesional corticomotor excitability in severe-stroke survivors (Table 2).

## 5. Combined rTMS and VR Training

Brain–computer interface (BCI) is one of the ways of improving neuroplasticity and providing sensor feedback on ongoing sensorimotor brain activities, allowing stroke survivors to self-modulate their sensorimotor brain activities. Three chronic stroke survivors were included in evaluating the effectiveness of combined rTMS+BCI versus sham-rTMS+BCI on motor recovery for three weeks (3×/week) [11]. The outcomes showed the viability and effectiveness of rTMS+BCI for motor recovery, as indicated by an increase in ipsilesional motor activity and improvements in behavioral function for the real rTMS+BCI condition, as well as the value of BCI-training alone shown by behavioral improvements in the sham rTMS+BCI condition. This demonstrates how rTMS treatment may enhance ipsilesional activation by modulating interhemispheric inhibition interactions.

Another 4-week treatment study assessing the effect of combined LF-rTMS and VR-training demonstrated a significant increase in FMA-UL, Wolf Motor Function Test, and Modified Barthel Index (MBI) in the experimental group compared to the control group. The results suggested that the combined use of LF-rTMS with VR training could effectively improve UL function, the activity of daily living, and quality of life and will provide better rehabilitation treatment for subacute stroke (Table 3). In order to restore movement, rehabilitation treatment helped create new motor-projection zones and arouse resting synapses to transfer nerve impulses. Although the mechanism is still unclear, it was supposed that rTMS might alter synaptic efficacy analogous to low-term potentiation and low-term depression [24]. Facilitating the use and enhancing motor performance in stroke survivors could be another possible mechanism of rTMS to restore a motor-function recovery [24].

Out of the four articles included in this section, two articles have used BCI along with VR/rTMS (Table 3). BCI technology can detect user intent even without corresponding motor output and may provide a meaningful form of feedback to the subjects before physical movement is possible. Success within the BCI task informs users that the brain state created was desirable, irrespective of any noticeable motor movement at the same time. Relying on the motor activity as an ideal output indicator (as performed in conventional training) could be discouraging during the initial recovery period, with patients having inherently limited active movement, whereas VR requires repetitive and intensive task practice in simulated environments with the active involvement of the affected limb along with performance feedback (visual, auditory, sensory).

The combination of VR and rTMS has been explored as an emerging potential therapeutic approach for stroke rehabilitation in this mini-review. VR technology has been used in stroke rehabilitation to create an interactive and immersive environment that provides feedback to the patient about their movements and engages them in task-specific exercises. rTMS, on the other hand, has been shown to be effective in modulating the activity of specific brain regions and promoting neural plasticity, which is crucial for stroke recovery. In our opinion, the combination of these two different modalities might be clinically helpful in several ways by synergizing their individual benefits in a single therapeutic protocol. The addition of a VR module might enhance the efficacy of training with the provision of better patient engagement, an enriched experience, and, hence, more adherence to therapy which is a crucial factor for recovery. rTMS can stimulate specific brain regions, while VR can provide performance feedback based on the patient’s movements and could facilitate better neuroplasticity. Finally, the combination can be helpful in providing a personalized approach to stroke rehabilitation by tailoring treatment to an individual’s specific needs and abilities. The use of both technologies can also enable therapists to track patient progress in a better quantitative way and adjust treatment plans accordingly with more customizable features. However, this is still an open area of research, and future studies with more clinical evidence are needed in this regard to realize the optimal potential of such combinations.

**Table 3:** Combined VR and rTMS studies primarily focusing on distal upper limb.

Sl No. and Studies	Participants	Device Used	Intervention	Outcome measures	Joints Involved	Findings
1. Chen et al. [93]	23 stroke survivors	VCT (Virtual Reality-based cycling training)	2 groups: (1) Sham iTBS+ VCT; (2) iTBS+VCT Each patient received iTBS or sham stimulation before the 60 min VCT program on the same day for 15 consecutive working days (3 weeks)	ARAT, FMA-UE, SIS, MAS-UE, MAL, 9HPT, BBT	Fingers, Wrist, and Elbow	↑ ARAT, FMA-UE in both groups ↑ SIS, MAS-UE, MAL, in iTBS+VCT
2. Sánchez-Cuesta et al. [12]	42 sub-acute stroke survivors	“NewROW” BCI-VR [94]	10 sessions rTMS	MI, FMA-UE, SIS, MAS, BI, FTT, 9HPT, RMT	Results to be published	Trial showed the additive value of VR immersive motor imagery as an adjuvant therapy combined with a known effective neuromodulation approach opening new perspectives for clinical rehabilitation protocols
3. Johnson et al. [11]	3 chronic stroke survivors	rTMS and BCI training was conducted using 64 channel tms compatible EEG caps along with BrainAmp MR Amplifier	3x/week, 3 weeks of combined real rTMS + BCI to one participant, Sham rTMS + BCI to another participant followed by BCI alone to third participant. [rTMS applied immediately prior to BCI training]	Outcome measure— Beck depression inventory, MMSE, FMA UL, MAS, EHI	Hand	↑↑ ipsilesional motor activity and improvement in behavioral function for the real rTMS + BCI group. Behavioral improvement demonstrated by for the sham rTMS + BCI condition
4. Zheng et al. [24]	112 stroke survivors	The BioMaste system (Jumho Electric Co., China). (Non-immersive)	2 groups (real LF-rTMS + VE, sham rTMS + VE); (30 min/session, 6 times/wk, total 24 sessions) VE started within 10 min of LF-rTMS; all participants provided with 30 min of PT, 30 min of OT, and 30 min of task practice in VE	Primary outcome—U-FMA, WMFT Secondary Outcome—MBI and SF-36	Shoulder, Elbow and Wrist	Significant ↑ in U-FMA, WMFT, MBI scores suggested the combined use of LF rTMS with VR training could effectively improve the upper limb function, the living activity, and the quality of life in patients with hemiplegia following subacute stroke

Abbreviations: 9HPT: Nine Hole Pegboard Test; ARAT: Action Reach Arm Test; BCI: Brain–Computer Interface; BI: Barthel Index; EHI: Edinburgh Handedness Inventory; EEG: Electroencephalogram; FMA-UE: Fugl–Meyer Assessment-Upper Extremity; FTT: Finger-Tapping Test; MAL: Motor Activity Log; MAS: Modified Ashworth Scale; MAS-UE: Modified Ashworth Scale Upper Extremity; iTBS: Intermittent Theta Burst Stimulation; LF-rTMS: Low Frequency Repetitive Transcranial Magnetic Stimulation; MI: Motricity Index of the Arm; MMSE: Mini-Mental State Examination; OT: Occupational Therapy; RMT: Resting Motor Threshold; rTMS: Repetitive Transcranial Magnetic Stimulation; SF-36: Short Form Survey; SIS: Stroke Impact Scale; VCT: Virtual Reality-Based Cycling Training; VR/VE: Virtual Reality/Virtual Environment; WMFT: Wolf Motor Function Test.

## 6. Perspective

This mini-review article identifies the novel emerging techniques, such as VR and rTMS, and emphasizes the potential of these new technologies in combination, holding promise for stroke rehabilitation. VR is seen as a potential tool for providing meaningful, realistic experiences and facilitating positive rehabilitation outcomes. Additionally, rTMS and motor-skill training using VR are novel treatment options in the field of neurorehabilitation that may further improve cortico-subcortical connectivity, leading to higher clinical effects regarding motor recovery. Various combinations which can be explored are HF-rTMS for enhancing ipsilesional hemispheric activity with VR and LF-rTMS for inhibiting contralesional hemispheric activity with VR. These intelligent rehabilitation technologies operate to capitalize on the inherent phenomena of brain plasticity. This capacity of the brain allows it to comply with change and environmental stimuli, such as neurological insults, therapeutics, and experiences, by modifying the brain’s anatomical structure, function, and neural connections [10]. A possible and more feasible combination could be home-based VR settings and rTMS in clinical settings.

Consequently, the pairing of VR therapy and rTMS-approach in rehabilitation solutions might have a positive impact on health infrastructure with respect to the optimization of professional resources. Furthermore, both therapies can be considered to magnify or complement the effect of the other, thereby attenuating the overall cortical reorganization in stroke survivors. Since VR has become a feasible rehabilitation tool, VR interventions have demonstrated their ability to provide patients with intensive, repetitive, and task-specific entrainment tools in naturalistic virtual environments. Recent studies have shown that stroke survivors’ upper limb motor function significantly improves when VR-based therapy and conventional therapy are combined. Beyond the evidence of their effectiveness, VR systems seem to provide patients with extremely engaging and motivating activities in virtually realistic worlds that may resemble the actual world. VR therapy focused on distal joints might be effective in improving the patient’s motor ability to perform the activity of daily living, such as opening jars, writing, and using utensils. In addition to rTMS, it can further enhance cortico-subcortical connectivity, leading to higher clinical effects regarding motor recovery. VR-based rehabilitation has been shown to improve occupational performance in stroke survivors by allowing them to engage in simulated real-life activities, providing a safe, controlled, and enriched environment for practicing and resulting in regaining functional independence, thus leading to improved occupational performance. Combining VR and rTMS interventions may have a synergistic effect on occupational performance, as both techniques target different aspects of rehabilitation. While VR can provide the opportunity to practice real-life activities in a controlled environment, rTMS can stimulate specific brain regions to enhance their ability to process and perform those activities. By incorporating these technologies into treatment plans, occupational therapists can provide more effective and engaging interventions for their patients. Additionally, these technologies can help improve the potential overall outcomes of occupational therapy by promoting greater recovery of motor function and enabling patients to better engage in the activity of daily living.

The authors strongly hypothesize that combining VR with augmented feedback and rTMS might have a promising potential for rehabilitation training. It is also important to note that several challenges are still to be addressed before its wider implementation in clinical settings, and it might not be feasible or appropriate to set up for all clinics or patients. Ultimately, the choice of therapy will depend on a range of factors, including patients’ specific needs and the resources available in the healthcare settings to be deployed. Careful consideration of the risks and benefits of each modality as well as the individual patient’s preferences, should be taken into account while making a decision about which therapy to pursue. It is equally important to consult with the concerned healthcare experts and technical professionals before making any decisions regarding the suitability and feasibility of implementing such therapies individually or in combination.

**Future Direction**: Standardized measurements of outcome variables of performance relevant for distal-UE also demands further investigation. Factors, such as the number of sessions, optimal stimulation parameters, temporal relationship, duration of VR and non-invasive brain stimulation therapies, and gaps between the therapies, still need to be explored. The combination of these potential strategies and the detailed objective evaluations of UL might lead to a new theory regarding how distinct neuromodulation approaches affect homeostatic plasticity and, as a result, motor recovery. Future studies could focus on developing some standard protocol for using such technologies which can be used across geographical barriers. Further studies are required on a larger cohort of patients with follow-up for evaluating any long-term benefits, but the results suggest that VR-based rehabilitation might be effective when combined with specific neurorehabilitation techniques for improving motor recovery. Large-scale future clinical trials are required to validate the synergistic effect and ability to induce clinically relevant neuroplasticity for the combination of the two, to enhance distal UL post-stroke.

While emerging technologies in rehabilitation, such as VR and rTMS, have demonstrated therapeutic potential in research settings, some of the barriers to entry factors should be addressed before their translation is effectively employed in routine clinical settings. The associated cost with emerging technologies, such as immersive VR and rTMS, including equipment, software, and the requirement for trained personnel to monitor such equipment, could be a potential barrier to implementing such technologies in resource-limited settings. Although reports on patient experiences are still scarce, insights from the patients’ perspectives may help researchers and healthcare professionals to identify topics that are important for patients undergoing a treatment, which could improve future trial design and subsequent clinical implementation. To be widely acceptable, any developed VR platform must be sensitive to socio-economic, cultural, and geographical barriers. In addition, patients’ expectations can provide important predictors of treatment outcomes [95]. Moreover, because of limited evidence of its therapeutic potential, it is challenging to justify the expenses of utilizing VR in such scenarios. The associated cyber-sickness, motion sickness, latency, and system malfunction might sometimes affect the end-user’s experience during prolonged usage. The use of VR technology in clinical practice may be subject to regulatory and ethical oversight, which can pose additional challenges in terms of compliance and approval processes. Stroke survivors having a significantly declined level of cognition having other disorders, such as epilepsy, seizures, etc., might not be suitable for undergoing VR-based training as it might pose a safety concern to their medical conditions.

Similarly, patients having metal implants, surgery, a history of epilepsy, or other progressive disorders might not be suitable for an rTMS-based treatment. Selection of a suitable patient cohort might be challenging and require a careful screening process to ensure safety guidelines. While rTMS is generally considered safe, individuals might have different levels of tolerability and might experience mild side effects such as headaches or discomfort during treatment [96]. More serious side effects, such as seizures, are rare but can occur in some patients. Because of the limited availability of specialized TMS centers and trained professionals, its access to remote areas is mostly restricted to resource-limited settings. There is a lack of standardized treatment protocols for rTMS-therapy in rehabilitation, and the optimal treatment parameters for different conditions are still being studied. This could make it difficult for healthcare providers to determine the most effective treatment protocol for their patients.

Being early-stage emerging technologies and an open area for research, the combination of two different modalities (VR and rTMS) might face some challenges, and that must be addressed to realize its full potential in clinical practice. The actual customization in protocol adjustment is a must for obtaining optimal therapeutic benefits for an individual, and therefore, rehabilitation professionals may require additional training to learn how to use and integrate these technologies into their treatment plans effectively. Some patients may not be comfortable with the combined use of VR and rTMS and may require additional education and support to accept these technologies as part of their treatment plan. The combined use of VR and rTMS in rehabilitation may be subject to regulatory barriers and approval processes, which can delay the implementation of these technologies.

Overall, the preliminary results are encouraging, but these technologies are still in the academic phase and require careful consideration and planning before they can be effectively translated into clinical practice. Large-scale future studies are required to address the associated barriers to make this practice widely acceptable across a wide range of patient cohorts. As technology continues to evolve, it is likely that some of these challenges will be overcome and will facilitate the widespread adoption of VR and rTMS (both individual and combined) in healthcare settings.

**Limitations:** Limited studies are available on VR and rTMS combinations for targeted distal UL rehabilitation. Although it shows promising results, there has been inconclusive evidence due to several limiting factors. First, the complexity of the study design, such as the discrepancies in different experimental techniques, methodologies, and duration of sessions. Second, small sample size, no control group, and heterogeneity in the stroke cohort, including age, chronicity, and extent and type of lesion. Third, a lack of better methods of functional change evaluation, such as functional MRI and PET, and a lack of multi-centric involvement and only short-term evaluations are described in the reviewed studies. Fourth, spontaneous recovery of motor function and exposure to other forms of additional rehabilitation therapies can act as confounding factors as well. Fifth, the lack of standardization of protocols and the use of diverse experimental techniques and methodologies leads to inconsistent results. In addition, VR protocols were described as having limited reproducibility due to the lack of details of a particular set of games in the entire set of video games, such as Nintendo Wii or Xbox. Next, the temporal application of VR and non-invasive brain stimulation techniques, such as gaps or required time differences between techniques, are neither fully described nor understood. Most studies consist of single sessions, compromising the attenuated effect that multiple sessions might have [48]. Most of the studies focused on distal UL have small sample sizes. This mini-review paper attempts to fill the gap in the literature for evolving new technologies, VR and rTMS, in stroke rehabilitation, and it was only focused on stroke, distal upper-limb, VR, and rTMS. Considering this to be an active and open area of research, an elaborate meta-analysis will be very beneficial for the larger community.

## Data Availability

Not applicable.

## Supplementary Materials

The following supporting information can be downloaded at: https://www.mdpi.com/article/10.3390/jcm12082944/s1.
