# Supplementary material for: Post-Stroke Rehabilitation of Distal Upper Limb with New Perspective Technologies: Virtual Reality and Repetitive Transcranial Magnetic Stimulation—A Mini Review
Source: J Clin Med. 2023 Apr 18;12(8):2944. doi: 10.3390/jcm12082944 (PMC10142518; doi:10.3390/jcm12082944)
Supplement: Supplementary file 1 [file jcm-12-02944-s001.zip › jcm-2316792-supplementary.pdf]

Post-Stroke Rehabilitation of Distal Upper Limb with New Perspective Technologies: Virtual Reality and Repetitive Transcranial Magnetic Stimulation—A Mini Review

Supplementary Material

A review article [1] considered conventional therapy, which included any activity designed to be therapeutic at the impairment, activity, or participation level that did not have the use of VR. In Supplementary Table 1, we have elaborated the objectives of the therapies used in some of the studies mentioned in the systematic review.

Supplementary Table S1: Description of conventional therapy Used in the studies cited in the systematic review

Sl. No.	Author	Description of Conventional Therapy Used
1.	Housman et al. [2]	Control activities consisted of self-range of motion (SROM) stretches and active range of motion (AROM) strengthening exercises throughout the hemiparetic upper extremity. During SROM stretches, participants clasped the hands or arms together and used the strength of the less-affected arm to move the affected arm through the available ROM at each joint. During AROM exercises, the hemiparetic arm was supported against gravity by a tabletop, and a towel was placed under the arm to decrease friction as subjects completed specified movements unilaterally. Additional activities consisted of using the affected arm as a functional assist during a prescribed list of activities of daily living (ADL) tasks (such as wiping a table or holding a container while the less-affected hand opened the lid) as well as hemiparetic upper extremity weight bearing on an open hand with the affected arm extended at the side of the body
2.	Shin et al. [3]	Standard occupational therapy involved range of motion and strengthening exercises for the affected limb, table- top activities, and training for ADL
3.	Kiper et al. [4]	The patients in the control group were asked to perform the exercises of postural control, exercises for hand pre-configuration, manipulative and functional skills exercises, proximal-distal exercises coordination with physiotherapist assistance and without it. The upper limb motions were trained with progressive complexity. To achieve the requested goal (in a horizontal or vertical plane) patients performed various movements, for example: shoulder flexion and extension, shoulder abduction and adduction, shoulder internal and external rotation and shoulder large circular movement, elbow flexion and extension, forearm pronation and supination, hand grasping-release and clenching into a fist. The rehabilitation programme was planned in accordance with the patients' current capacity.
4.	Levin et al. [5]	Conventional therapy consisted of occupational therapy, including exercises involving reaching for and holding cones, cups, and other objects in all motion planes with and without external loading.

References

1. Domínguez-Téllez, P.; Moral-Muñoz, J.A.; Salazar, A.; Casado-Fernández, E.; Lucena-Antón, D. Game-Based Virtual Reality Interventions to Improve Upper Limb Motor Function and Quality of Life after Stroke: Systematic Review and Meta-Analysis. *Games Health J.* **2020**, *9*, 1–10.

2. Housman, S.J.; Scott, K.M.; Reinkensmeyer, D.J. A Randomized Controlled Trial of Gravity-Supported, Computer-Enhanced Arm Exercise for Individuals with Severe Hemiparesis. *Neurorehabil. Neural Repair* **2009**, *23*, 505–514.

3. Shin, J.-H.; Kim, M.-Y.; Lee, J.-Y.; Jeon, Y.-J.; Kim, S.; Lee, S.; Seo, B.; Choi, Y. Effects of Virtual Reality-Based Rehabilitation on Distal Upper Extremity Function and Health-Related Quality of Life: A Single-Blinded, Randomized Controlled Trial. *J. Neuroeng. Rehabil.* **2016**, *13*, 17, doi:10.1186/s12984-016-0125-x.

4. Kiper, P.; Piron, L.; Turolla, A.; Stożek, J.; Tonin, P. The Effectiveness of Reinforced Feedback in Virtual Environment in the First 12 Months after Stroke. *Neurol. Neurochir. Pol.* **2011**, *45*, 436–444.

5. Levin, M.F.; Snir, O.; Liebermann, D.G.; Weingarden, H.; Weiss, P.L. Virtual Reality versus Conventional Treatment of Reaching Ability in Chronic Stroke: Clinical Feasibility Study. *Neurol. Ther.* **2012**, *1*, 1–15.
